# Docking interactions determine substrate specificity of members of a widespread family of protein phosphatases

**DOI:** 10.1016/j.jbc.2024.107700

**Published:** 2024-08-22

**Authors:** Suhaily Caban-Penix, Kristin Ho, Zhewen Yang, Rishika Baral, Niels Bradshaw

**Affiliations:** 1 Molecular and Cell Biology Program, Brandeis University, Waltham, Massachusetts, USA; 2 Department of Biochemistry, Brandeis University, Waltham, Massachusetts, USA; 3 Biochemistry and Biophysics Program, Brandeis University, Waltham, Massachusetts, USA

**Keywords:** protein phosphatase, PPM phosphatase, *Bacillus subtilis*, substrate specificity, sporulation, general stress response, STAS domain, anti-sigma factor, alternative-sigma factor

## Abstract

How protein phosphatases achieve specificity for their substrates is a major outstanding question. PPM family serine/threonine phosphatases are widespread in bacteria and eukaryotes, where they dephosphorylate target proteins with a high degree of specificity. In bacteria, PPM phosphatases control diverse transcriptional responses by dephosphorylating anti-anti-sigma factors of the STAS domain family, exemplified by *Bacillus subtilis* phosphatases SpoIIE, which controls cell-fate during endospore formation, and RsbU, which initiates the general stress response. Using a combination of forward genetics, biochemical reconstitution, and AlphaFold2 structure prediction, we identified a conserved, tripartite substrate docking interface comprised of three variable loops on the surface of the PPM phosphatase domains of SpoIIE and RsbU that recognize the three-dimensional structure of the substrate protein. Nonconserved amino acids in these loops facilitate the accommodation of the cognate substrate and prevent dephosphorylation of the noncognate substrate. Together, single-amino acid substitutions in these three elements cause an over 500-fold change in specificity. Our data additionally suggest that substrate-docking interactions regulate phosphatase specificity through a conserved allosteric switch element that controls the catalytic efficiency of the phosphatase by positioning the metal cofactor and substrate. We hypothesize that this is a generalizable mechanistic model for PPM family phosphatase substrate specificity. Importantly, the substrate docking interface with the phosphatase is only partially overlapping with the much more extensive interface with the upstream kinase, suggesting the possibility that kinase and phosphatase specificity evolved independently.

Signaling by reversible phosphorylation requires that opposing kinases and phosphatases have exquisite specificity for their respective substrate proteins (1, 2, 3). While the mechanisms of kinase specificity, in which sequences surrounding the phosphorylation site dock into a deep active site groove, are well understood (4), much less is known about how phosphatases discriminate between substrates (3). Here, we address the mechanism of how phosphatases achieve substrate specificity with two bacterial serine/threonine phosphatases of the PPM family, SpoIIE and RsbU from *B. subtilis*, that must discriminate between their respective substrate proteins in a biological context.

Precisely regulated serine/threonine phosphatases of the PPM family are widespread regulators of bacterial transcriptional responses (5, 6). Many organisms have multiple phosphatases that must discriminate between related substrate proteins to maintain signaling fidelity, but the molecular mechanisms of substrate recognition and specificity are not understood (6, 7, 8, 9). A particular challenge to determining how PPM phosphatases achieve specificity is that the active site is accessible on the solvent-exposed surface. Here, we determine the mechanism of specificity for two phosphatases from *Bacillus subtilis*. SpoIIE dephosphorylates SpoIIAA to specify cell fate during endospore formation by activating σ^F^ (10, 11), and RsbU dephosphorylates RsbV to initiate the general stress response by activating σ^B^ (12) (Fig. 1*A*). SpoIIAA and RsbV are paralogs that share the STAS domain fold and share 29 percent sequence identity (13, 14, 15, 16).

**Figure 1:**
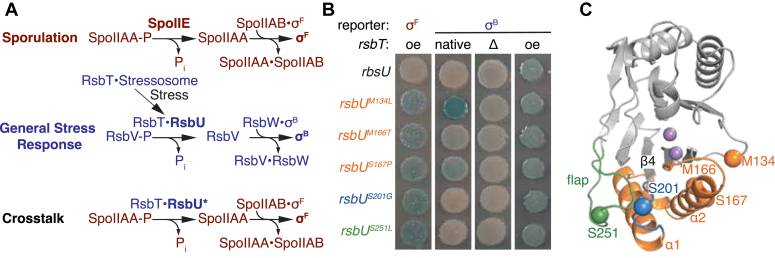
**Isolated crosstalk mutants fall into two phenotypic classes.***A*, a depiction of the sporulation (*red*) and general stress response pathways (*blue*), along with the premise for our genetic screen in looking for crosstalk mutants. PPM phosphatases, SpoIIE (*red*), and RsbU (*blue*) dephosphorylate paralogous substrates SpoIIAA (*red*) and RsbV (*blue*), respectively. Activation of RsbU is dependent on RsbT. Dephosphorylation of SpoIIAA and RsbV activates a partner-switching mechanism, where the unphosphorylated substrates SpoIIAA and RsbV bind to the kinases SpoIIAB (*red*) and RsbW (*blue*). The binding of the substrates to the kinases releases the sigma factors σ^F^ (*red*) and σ^B^ (*blue*), while phosphorylation of SpoIIAA by SpoIIAB and RsbV by RsbW resets the system. A screen was developed to isolate RsbU crosstalk mutants that could activate σ^F^ in a strain that was deleted for *spoIIE*. *B*, RsbU variants with amino acid substitutions (M134L, M166T, S167P, S201G, and S251L) isolated in the genetic screen activate σ^F^ and retain their ability to activate σ^B^ in *B. subtilis*. Reporter strains with *lacZ* under the control of either the σ^F^ (*left*) or σ^B^ (*right*) promoter were plated on indicator plates containing X-gal and IPTG. The first σ^B^ reporter strain has *rsbT* on the chromosome (*left*), the second *rsbT* is deleted (*middle*), and the last *rsbT* is overexpressed (oe) from plasmid pHB201. Plates were imaged after 24 h of growth at 37 °C. *C*, AlphaFold2 structure of RsbU phosphatase domain. Residues M166 and S167 are found in the switch (*orange*), which includes the α1 and α2 helixes. M134 (*orange*) is adjacent to the switch. S201 (*blue*) is in the loop between α1(*orange*) helix and β4 (*gray*). S251 is located in the flap (*green*). The two metals sit at the catalytic center (*purple*). IPTG, isopropyl-beta-D-thiogalactoside; X-gal, 5-bromo-4-chloro-3-indolyl-beta-D-galacto-pyranoside.

Three features of these systems make them ideal for understanding molecular mechanisms of phosphatase specificity. First, crosstalk between these pathways is highly detrimental; activation of σ^B^ by SpoIIE blocks sporulation (17), and activation of σ^F^ by RsbU causes lethality (Fig. S1). Second, each phosphatase acts on a single phosphoserine on a single substrate protein, simplifying the analysis of changes in specificity in both cellular and biochemical contexts (12, 18, 19). Third, we have previously biochemically reconstituted the specificity of both phosphatases and have found them to be highly specific (SpoIIE has an approximately 4000-fold greater k_cat_/K_M_ for SpoIIAA and RsbU has approximately an 400-fold greater k_cat_/K_M_ for RsbV) (8).

An important consideration for phosphatase specificity is that every substrate of a protein-phosphatase is shared with an opposing kinase. An unanswered question is whether the kinases and phosphatases recognize the same or different features of their shared substrate proteins. Whether the same features of the substrate proteins are recognized by both enzymes determines how changes in substrate sequence impact signaling and constrain the pathways available for evolving new signaling functions. SpoIIAA and RsbV are each phosphorylated by a cognate kinase/anti-sigma factor (SpoIIAB and RsbW, respectively) that undergoes a partner-switch to release the sigma factor when the substrate is dephosphorylated (12, 20) (Fig. 1*A*). X-ray crystal structures revealed extensive interfaces surrounding the phosphorylation sites for both SpoIIAA/SpoIIAB and RsbV/RsbW complexes, enabling direct comparison of how the phosphatases and kinases recognize their substrates (15, 21).

PPM family phosphatases use two divalent cations in their active sites to deprotonate a water that is the nucleophile for attack of the phospho-serine (3, 22). Our structural and biochemical studies revealed that the activity of SpoIIE and RsbU is controlled by the conformational change of an α-helical switch element at the base of the phosphatase domain (α1 and α2) that coordinates the metal cofactor (6, 8, 9). Subsequent genetic and biochemical experiments implicated this element in substrate specificity, but the molecular mechanistic basis for this was not known (8). One clue is that a variable insertion region termed the “flap” that has been implicated in other systems packs against the switch element, suggesting that substrate docking could be transmitted through these contacts (9, 23, 24, 25, 26). However, the binding interface of the substrate protein and phosphatase had not been identified.

Using a combination of genetics, biochemical reconstitution, and AlphaFold2 structure prediction, we have discovered the molecular basis for how SpoIIE and RsbU specifically recognize their respective substrate proteins. They use a conserved tri-partite binding site where the folded protein substrate engages with three variable loops of the phosphatase domain that dock against the switch element to position the substrate and form the catalytic center. We hypothesize that this is a broadly generalizable mechanism by which PPM family phosphatases engage with and achieve specificity for their substrates.

## Results

### Isolation of RsbU crosstalk mutants

To identify the features of RsbU that determine substrate specificity, we designed a genetic screen to isolate crosstalk variants of RsbU that dephosphorylate the off-pathway substrate, SpoIIAA, and activate σ^F^ (Fig. 1*A*). We introduced plasmids with PCR mutagenized *rsbT* and *rsbU* genes to a σ^F^ reporter strain lacking the σ^B^ operon (including *rsbT* and *rsbU*) and *spoIIE*, the phosphatase responsible for activating σ^F^. We then screened for plasmids that cause activation of σ^F^ under sporulation conditions when *rsbT* and *rsbU* are expressed. Performing the screen under sporulation conditions was essential because uncompartmentalized activation of σ^F^ is lethal (Fig. S1). Because expression of the SpoIIA operon, which includes σ^F^, occurs only in a subpopulation of cells during sporulation, this allows isolation of cells carrying plasmids that drive improper activation of σ^F^. After an initial round of screening did not yield any crosstalk mutations, we increased the sensitivity of our screen by decreasing the activity of the SpoIIAB kinase (using a strain with *spoIIAB*^*R105C*^) (27). We identified five *rsbU* mutants that caused robust σ^F^ activation from this screen (M134L, M166T, S167P, S201G, and S251L) but did not isolate mutations in *rsbT* (Fig. 1*B*). All amino acid substitutions mapped to the phosphatase domain of RsbU. While two *rsbU* variants were only isolated from one pool of mutagenized plasmids, the others were picked up from two or more independently generated plasmid pools, suggesting that the screen was near saturation.

### Crosstalk mutants fall into two phenotypic classes

There are three possible models for how *rsbU* crosstalk mutations cause σ^F^ activation in our screen. First, crosstalk mutations could swap RsbU specificity, increasing activity toward SpoIIAA and decreasing activity toward RsbV. Second, crosstalk mutations could cause indiscriminate dephosphorylation of both SpoIIAA and RsbV. Third, crosstalk mutations could hyperactivate RsbU, leading to SpoIIAA dephosphorylation without a change in specificity. To qualitatively distinguish between these models, we transformed plasmids containing rebuilt versions of the *rsbU* crosstalk mutants into additional reporter strains.

First, to determine whether any RsbU variants lost activity toward RsbV, we overexpressed the *rsbU* variants in a σ^B^ reporter strain deleted for *rsbTU* on the chromosome. All five *rsbU* mutants robustly activated σ^B^ similar to wildtype *rsbU*, indicating that they retain activity toward RsbV and are at least somewhat promiscuous (Fig. 1*B*).

Second, to determine whether any of the RsbU mutants had increased activity toward RsbV, we rebuilt the *rsbU* mutations in a plasmid that did not contain *rsbT* and transformed these plasmids into σ^B^ reporter strains. *rsbU*^*M134L*^, *rsbU*^*M166T*^, and *rsbU*^*S167P*^ activated σ^B^ in a strain background where *rsbT* was present on the chromosome, while none of the *rsbU* variants activated σ^B^ in a strain deleted for *rsbT* (Fig. 1*B*). When RsbT is sequestered in the stressosome, wildtype RsbU does not sufficiently dephosphorylate RsbV to activate σ^B^ in the absence of stress. Therefore, activation of σ^B^ in this context (either with or without rsbT on the chromosome) is indicative of hyperactivity. From this, we conclude that the *rsbU* variants fall into two classes: M134L, M166T, and S167P are hyperactivating, while S201G and S251L are not.

### A structural model for phosphatase/substrate interaction

To further characterize the RsbU crosstalk variants, we mapped their locations onto the RsbU phosphatase domain from an AlphaFold2 model that we generated of dimeric RsbU (6) (Fig. 1*C*). The mutations cluster in two regions:

M134, M166, and S167 form a cluster, buried in the core of the phosphatase domain around the α1 helix (Fig. 1*C*). The α1 and α2 helices control phosphatase activation and substrate recognition in the paralogous phosphatase, SpoIIE, suggesting that this mechanism is conserved with RsbU (6, 8, 9). Interestingly, we previously isolated substitutions at M166 in a screen to identify RsbT-independent variants of RsbU (8) and in a suppressor screen to restore activity to an RsbU variant that has reduced binding to RsbT (6). To determine whether the identity of the amino acid substitution at M166 differentially impacted σ^F^ and σ^B^ activity, we generated an allelic series replacing M166 with amino acids of varied size and hydrophobicity. The four variants (M166T, V, L, and I) that activated σ^F^ also activated σ^B^ in the absence of stress, suggesting that hyperactivity and promiscuity are related (Fig. S2*A*).

**Figure 2:**
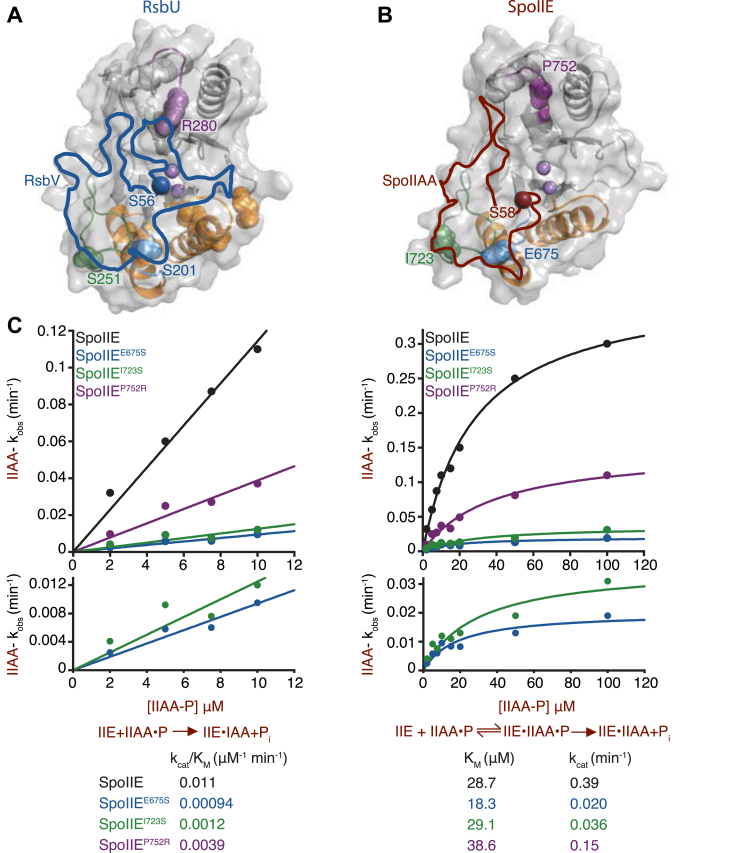
**Conservation at the phosphatase/substrate interface positions SpoIIAA for efficient catalysis.***A*, Alphafold2 structure of RsbU phosphatase domain. The position of RsbV (*blue*) at the RsbU/RsbV interface is outlined based on a 1.4 Å probe radius with a transparent surface model shown. RsbV phosphorylation site, S56 (*red*) sites near the catalytic center where the two metals are coordinated (*light purple*). Residue R280 is located in the α3/4 loop. *B*, Alphafold2 structure of SpoIIE phosphatase domain. The position of SpoIIAA at the SpoIIE/SpoIIAA interface is outlined (*red*) with a 1.4 Å probe radius. SpoIIAA phosphorylation site, S58 (*red*), is located near the catalytic center in proximity to the two metal cofactors (light purple). Residues E675S (*blue*), I723 (*green*), and P752 (*purple*) are located within the α1/β4 loop, flap, and α3/4 loop, respectively. *C*, graphs showing the rate of SpoIIE (*black*), SpoIIE^E675S^ (*blue*), SpoIIE^I723S^ (*green*), and SpoIIE^P752R^ (*purple*) dephosphorylation as a function of SpoIIAA-P concentration. The plot on the *left* displays the k_cat_/K_M_ values of SpoIIAA-P dephosphorylation by SpoIIE (*black*), SpoIIE^E675S^ (*blue*), SpoIIE^I723S^ (*green*), and SpoIIE^P752R^ (*purple*) with concentrations of SpoIIAA-P below the K_M_ fit to the linear equation (in KaleidaGraph) (k_cat_/K_M_)∗[SpoIIAA-P]. The k_cat_/K_M_ were SpoIIE 0.011 ± 0.00045 μM^–1^ min^–1^, SpoIIE^E675S^ 0.001 ± 7.0e^–5^ μM^–1^ min^–1^, SpoIIE^I723S^ 0.001 ± 0.00016 μM^–1^ min^–1^, and SpoIIE^P752R^ 0.0039 ± 0.00028 μM^–1^ min^–1^. The plot on the *right* was fit to the Michaelis-Menten equation (in KaleidaGraph) k_cat_∗[SpoIIAA-P]/(K_M_ + [SpoIIAA-P]). The k_cat_ for each enzyme were SpoIIE 0.39 ± 0.017 min^–1^, SpoIIE^E675S^ 0.020 ± 0.0026 min^–1^, SpoIIE^I723S^ 0.036 ± 0.0058 min^–1^, and SpoIIE^P752R^ 0.15 ± 0.014 min^–1^. The K_M_ measured for each enzyme were SpoIIE 28.7 ± 2.8 μM, SpoIIE^E675S^ 18.3 ± 6.0 μM, SpoIIE^I723S^ 29.1 ± 10.5 μM, and SpoIIE^P752R^ 38.6 ± 7.9 μM. The error is the error of the fit. Reactions were multiple turnover reactions with varying concentrations of SpoIIAA-P, 0.1 μM SpoIIE, 10 mM MgCl_2_, and 0.1 μM SpoIIAA-P^32^. Below each graph is a summary of the reaction. On the *left* are the kinetic parameters for the k_cat_/K_M_ reaction scheme, P_i_ indicating product, and k_obs_ values below. On the *right*, the kinetic scheme is used to summarize the parameters and values for k_cat_ and K_M_ values.

S201 and S251 are located on the opposite side of the active site from M166 and are predicted to be solvent-exposed, suggesting that they could make direct contact with RsbV (Fig. 1*C*). S201 is in the loop between α2 and β4, while S251 is located in a variable element of the phosphatase domain (referred to as the “flap”) that has been implicated in substrate recognition in other PPM phosphatases (9, 23, 24, 25, 26). This conclusion is supported by our AlphaFold2 model of a heterotetrameric RsbU/V complex that places S201 and S251 at the RsbU/V interface (Fig. 2*A*). The phosphorylation site, S56, is modeled near the catalytic center of RsbU, additionally supporting the validity of the model. RsbV is predicted to exclusively contact the phosphatase domain of RsbU and would not contact RsbT in the hetero-heptameric RsbT/U/V signaling complex. Thus, we conclude that S201 and S251 are likely to be directly involved in substrate recognition.

### Conservation of the phosphatase/substrate interface

Next, to assess whether the RsbU/V interface is shared with SpoIIE and SpoIIAA, we generated a similar AlphaFold2 model of a heterotetrameric SpoIIE/AA complex based on the dimeric structure of SpoIIE that we determined previously (9) (Fig. 2*B*). The model places SpoIIAA in a very similar position relative to the SpoIIE phosphatase domain as we observed in the RsbU/V complex (Fig. 2*A*). Importantly, SpoIIE residues corresponding to crosstalk variants RsbU^S201^ (SpoIIE^E675^) and RsbU^S251^ (SpoIIE^I723^) are buried in the interface. Additionally, both of these residues stand out as being variable elements of the contact interface, which is otherwise relatively conserved (Fig. S2*B*). One notable difference between the models is that SpoIIAA makes contacts, distant from the phosphorylation site, with the regulatory domain of SpoIIE (Fig. S2*B*). These contacts provide an explanation for why mutation of an amino acid in this interface (glutamine 73 to alanine, SpoIIAA^Q73A^) causes hyperactivation of σ^F^ (27) and further supports the validity of the structural model.

### Flap and switch loops position SpoIIAA for dephosphorylation

Next, we biochemically assayed how the contacts identified by our genetic screen and structural models determine substrate specificity. We used SpoIIE for these studies because we have more extensively studied SpoIIE specificity compared to RsbU, and the K_M_ of RsbU is below 1 μM for both SpoIIAA and RsbV, making measurement of k_cat_/K_M_ more challenging. We generated variants of the phosphatase domain of SpoIIE (SpoIIE^590-827^, which we previously found is sufficient to recapitulate substrate specificity (8)) that were substituted for the corresponding amino acid of RsbU at positions E675 (serine) and I723 (serine). Other than these nonconserved interface residues that were genetically identified, we selected one additional nonconserved interface residue to mutate based on analysis of conservation of the phosphatase/substrate interface predictions, SpoIIE^P752R^ (Figs. 2, *A* and *B*, S2*C*). P752 repositions a variable loop above the phosphatase active sites (RsbU^279-285^/SpoIIE^751-754^) that forms a contact with RsbV in the RsbU/RsbV complex model. We therefore hypothesized that the SpoIIE^P752R^ mutation might favor recognition of RsbV.

Using an assay that monitors dephosphorylation of ^32^P-SpoIIAA, we found that SpoIIE^E675S^ (k_cat_^SpoIIAA^/K_M_^SpoIIAA^ 0.001 μM^-1^ min^–1^) and SpoIIE^I723S^ (k_cat_^SpoIIAA^/K_M_^SpoIIAA^ 0.001 μM^–1^ min^–1^) had 10-fold reductions in k_cat_^SpoIIAA^/K_M_^SpoIIAA^ compared to SpoIIE (0.011 μM^–1^ min^–1^) (Fig. 2*C*). Extending these data to near saturating concentrations of SpoIIAA revealed that the primary effect of the substitutions was on the k_cat_^SpoIIAA^ and that there was no significant change in K_M_^SpoIIAA^ (Fig. 2*C*). Consistent with the defects in phosphatase activity being substrate specific, we observed no change in the activity toward the generic small-molecule substrate, p-nitrophenyl phosphate (Fig. S3). Thus, we conclude that variable positions in the flap and switch regions mediate substrate contacts important for achieving maximal catalytic efficiency for the cognate substrate.

The specificity and activity of SpoIIE and RsbU is additionally determined by recruitment of metal cofactor (8, 9). Thus, we held the substrate concentration constant and measured SpoIIE activity as a function of metal concentration (Fig. S4). None of the variants had a significant change in metal concentration dependence of activity, establishing that they do not impact this step of the reaction. We additionally explored the metal cofactor preference of the SpoIIE variants. Our initial assays were performed with magnesium, which we presume is the physiologically relevant metal. However, the phosphatase domain construct of SpoIIE is more active with manganese than magnesium, so we assayed SpoIIE^E675S^, one of the variants with the largest effects with manganese as the metal cofactor. In this case, we observed only a 2-fold decrease in activity, suggesting that the identity of the metal cofactor influences the effect of specificity determinants (Fig. S5). Together, we conclude that specificity determinants in the flap and switch regions support efficient catalysis of cognate substrate once bound in a manner that depends on the identity of the metal cofactor.

### The switch and α3/4 loop discriminate against RsbV

**Figure 3:**
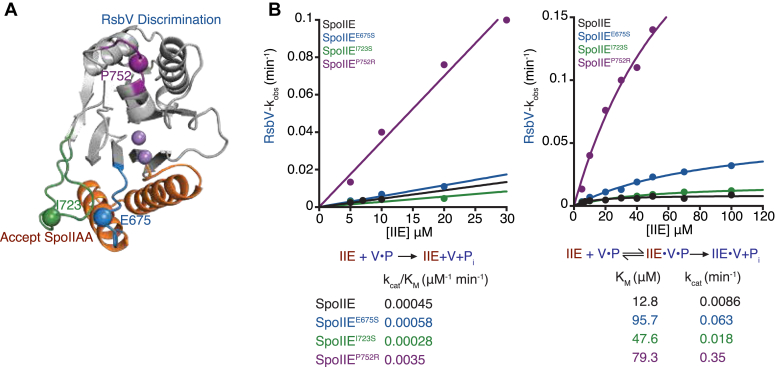
**The α3/4 loop contributes to specificity by discriminating against RsbV.***A*, AlphaFold2 structure of SpoIIE phosphatase domain. The SpoIIE phosphatase domain shows the positions of residues I723 (*green*), E675S (*blue*), P752 (*purple*), and metal cofactors (*light purple*) depicted on the structure. The rejection of RsbV (*blue*) by the residue P752 and acceptance of SpoIIAA (*red*) by residues I723S and E675 are noted on the structure. *B*, graphs showing the rate of SpoIIE, SpoIIE^E675S^, SpoIIE^I723S^, and SpoIIE^P752R^ dephosphorylation of RsbV-P. The plot on the *right* displays the k_cat_/K_M_ values of RsbV-P dephosphorylation by SpoIIE (*black*), SpoIIE^E675S^ (*blue*), SpoIIE^I723S^ (*green*), and SpoIIE^P752R^ (*purple*) with concentrations of RsbV-P below the K_M_ fit to a linear equation (in KaleidaGraph) (k_cat_/K_M_)∗[RsbV-P]. The k_cat_/K_M_ were SpoIIE 0.00045 ± 2.9e-5 μM^–1^ min^–1^, SpoIIE^E675S^ 0.00058 ± 3.8e^–5^ μM^–1^ min^–1^, SpoIIE^I723S^ 0.00028 ± 6.5e-5 μM^–1^ min^–1^, and SpoIIE^P752R^ 0.0035 ± 0.00028 μM^–1^ min^–1^.The plot on the *left* was fit to the Michaelis–Menten equation (in KaleidaGraph) kcat∗[RsbV-P]/(K_M_ + [RsbV-P]). The k_cat_ for each enzyme was SpoIIE 0.0086 ± 0.00080 min^–1^, SpoIIE^E675S^ 0.063 ± 0.0071 min^–1^, SpoIIE^I723S^ 0.018 ± 0.0021 min^–1^, and SpoIIE^P752R^ 0.15 ± 0.096 min^–1^. The K_M_ measured for each enzyme were SpoIIE 12.8 ± 4 μM, SpoIIE^E675S^ 95.7 ± 17.5 μM, SpoIIE^I723S^ 47.6 ± 12.0 μM, and SpoIIE^P752R^ 79.3 ± 31.6 μM. The error is the error of the fit. Reactions were single turnover reactions with varying concentrations of SpoIIE, 0.15 μM RsbV-P, 10 mM, and MnCl_2_. Below each graph is a summary of the reaction. On the *left* are the kinetic parameters for the k_cat_/K_M_ reaction scheme, P_i_ indicating product, and k_obs_ values below. On the *right*, the kinetic scheme is used to summarize the parameters and values for k_cat_ and K_M_ values.

Specificity is determined by the relative k_cat_/K_M_ for two competing substrates. Thus, to determine the contributions of the switch loop (SpoIIE-E675), flap (SpoIIE-I723), and α3/4 loop (SpoIIE-P752) to specificity, we measured hydrolysis of ^32^P-RsbV by SpoIIE variants. We conducted these assays under single-turnover conditions with manganese as the metal cofactor because the rate of hydrolysis with magnesium was too slow to accurately measure (8). Although we do not directly compare single- and multiple-turnover reaction rates, we expect the values of k_cat_ and K_M_ from each reaction setup to be equivalent for the following reasons: No lag phase was observed in any reaction, rendering unlikely the existence of any kinetically relevant intermediates following formation of the enzyme–substrate complex. In the cases for which we have measured both single and multiple turnover reactions, they have given indistinguishable results, indicating there are not likely to be rate determining steps after chemistry. Finally, product inhibition is not relevant in our multiple-turnover experiments because we measure initial velocities (8). We found that SpoIIE^P752R^ (k_cat_^RsbV^/K_M_^RsbV^ 0.0035 μM^–1^ min^–1^) was 17-fold more active toward RsbV-P than SpoIIE (k_cat_^RsbV^/K_M_^RsbV^ 0.00045 μM^–1^ min^–1^), while SpoIIE^E675S^ (k_cat_^RsbV^/K_M_^RsbV^ 0.00058 μM^–1^ min^–1^) and SpoIIE^I723S^ (k_cat_^RsbV^/K_M_^RsbV^ 0.00028 μM^–1^ min^–1^) did not significantly change activity (Fig. 3*B*). Extending the data to higher concentrations of SpoIIE demonstrated that the P752R substitution primarily increases the k_cat_^RsbV^ without changing the K_M_^RsbV^ (although we were not able to saturate the reaction due to insolubility of SpoIIE^P752R^ at high concentrations). We additionally observed some increase in k_cat_^RsbV^ for SpoIIE^E675S^ and SpoIIE^I723S^, but these effects were offset by increases in K_M_^RsbV^. Of note, we postulate that the magnitude of the effect could be an underestimate because our experiments with SpoIIAA suggest that SpoIIE is more promiscuous when manganese is used as the metal cofactor compared to magnesium. We conclude that the α3/4 loop is important for substrate discrimination and that the P752R substitution allows SpoIIE to accommodate the noncognate substrate, RsbV-P, in a manner more favorable for catalysis (Fig. 3*A*).

### Combinatorial control of substrate discrimination

**Figure 4:**
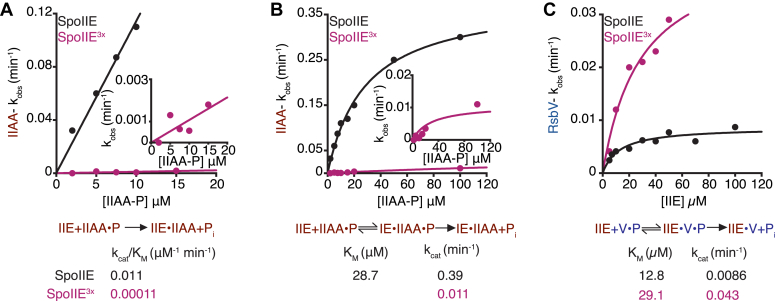
**Combination of all three amino acid substitutions substantially reduces substrate specificity**. All plots show the rates of SpoIIAA-P dephosphorylation by SpoIIE and SpoIIE^3x^ as a function of SpoIIAA-P concentration. *A*, the k_cat_/K_M_ values SpoIIAA-P dephosphorylation by SpoIIE (*black*) and SpoIIE^3x^ (triple mutation of E675S, I723S, and P752R, *pink*) with concentrations of SpoIIAA-P below the K_M_ were fit to the linear equation (in KaleidaGraph) (k_cat_/K_M_)∗[SpoIIAA-P]. The k_cat_/K_M_ was SpoIIE 0.011 ± 0.00045 μM^–1^ min^–1^ andSpoIIE^3x^ 0.00011 ± 2.4e^–5^ μM^–1^ min^–1^. The error is the error of the fit. Reactions were multiple turnover reactions with varying concentrations of SpoIIAA-P, 0.1 μM SpoIIE, 10 mM MgCl_2_, and 0.1 μM SpoIIAA-P^32^. Below the graph is a summary of the reaction scheme depicting the kinetic parameters for k_cat_/K_M_, P_i_, the product, and k_obs_ values below. *B*, rate of SpoIIAA-P dephosphorylation by SpoIIE (*black*) and SpoIIE^3x^ (*pink*) as a function of SpoIIAA-P concentration. The plot was fit to the Michaelis-Menten equation (in KaleidaGraph) k_cat_∗[SpoIIAA-P]/(K_M_ + [SpoIIAA-P]) using a nonlinear curve fitting. The k_cat_ measured was SpoIIE 0.39 ± 0.017 min^–1^ and SpoIIE^3x^ 0.011 ± 0.0018 min^–1^. The error is the error of the fit. The K_M_ for SpoIIE was 0.39 ± 2.8 μM. Reactions were multiple turnover reactions with varying concentrations of SpoIIAA-P, 0.1 μM SpoIIE, 10 mM MgCl_2_, and 0.1 μM SpoIIAA-P^32^. Below the graph is a summary of the reaction scheme depicting the kinetic parameters for k_cat_, K_M_, P_i_, the product, and k_obs_ values below. *C*, rate of RsbV dephosphorylation by SpoIIE (*black*) and SpoIIE^3x^ (*pink*) as a function of SpoIIE concentration. The plot was fit to a Michaelis-Menten equation (in KaleidaGraph) k_cat_∗[SpoIIAA-P]/(K_M_ + [SpoIIAA-P]) using a nonlinear curve fitting. The k_cat_ measured was SpoIIE 0.0086 ± 0.00080 min^–1^ and SpoIIE^x3^ 0.043 ± 0.0084 min^–1^. The K_M_ measured was SpoIIE 12.8 ± 4.0 μM and SpoIIE^x3^ 29.1 ± 11.8 μM. The error is the error of the fit. Reactions were single turnover reactions with varying concentrations of SpoIIE, 0.15 μM RsbV-P, 10 mM, and MnCl_2_. Below the graph is a summary of the reaction scheme depicting the kinetic parameters for k_cat_, K_M_, P_i_, the product, and k_obs_ values below.

Together, we identified three specificity determinants in the phosphatase domain of SpoIIE that recognize SpoIIAA (the switch-loop and flap) and reject RsbV (the α3/4 loop). To determine how these features work together, we generated a triple-mutant variant that combines all three substitutions (SpoIIE^3X^). For this variant, we observed a combinatorial effect, with a hundred-fold reduction in k_cat_^SpoIIAA^/K_M_^SpoIIAA^ (Fig. 4, *A* and *B*, S4), and a roughly 5-fold increase in k_cat_^RsbV^/K_M_^RsbV^ (Fig. 4*C*). Thus, these three specificity determinants combinatorially reduce the activity toward the cognate substrate while increasing the activity toward the noncognate substrate. Sequence alignments of diverse bacterial phosphatases reveal that these three loop elements are variable in sequence and length, supporting a model that they are shared determinants of substrate specificity.

### Substrate complementarity drives phosphatase specificity

**Figure 5:**
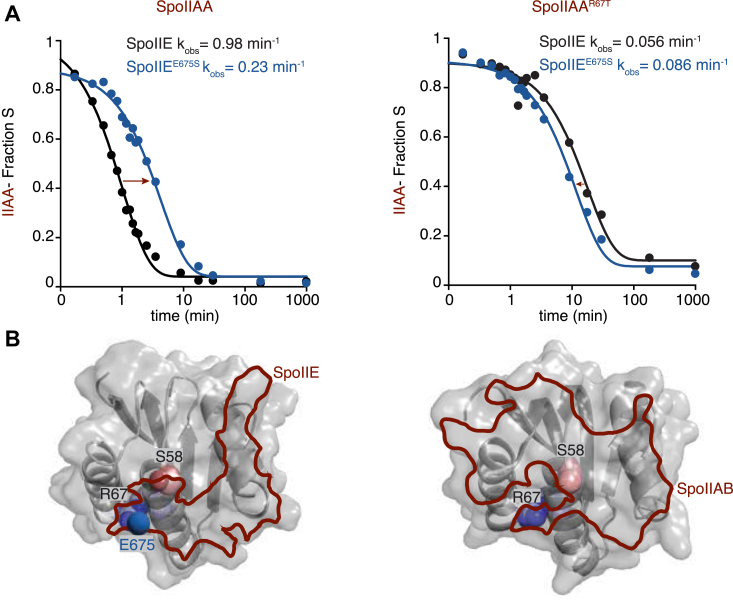
**Location of substrate residue complements phosphatase specificity**. *A*, rate of SpoIIAA-P and SpoIIAA^R67T^ dephosphorylation by SpoIIE and SpoIIE^E675S^ over time. Plot on the *left* measures the fraction of SpoIIAA-P dephosphorylated over time by SpoIIE (*black*) and SpoIIE^E675S^ (*blue*) and was fit to an exponential decay function. The measured k_obs_ were SpoIIE 0.98 ± 0.05 min^–1^ and SpoIIE^E675S^ 0.23 ± 0.015 min^–1^. The *right* plot measures the fraction of SpoIIAA^R67T^ dephosphorylated over time by SpoIIE (*gray*) and SpoIIE^E675S^ (*light blue*) and were fit to an exponential decay function. The k_obs_ was SpoIIE 0.056 ± 0.0061 min^–1^ and SpoIIE^E675S^ 0.086 ± 0.0062 min^–1^. Reactions were single-turnover reactions using 0.1 μM SpoIIE, 0.5 μM SpoIIAA-P, 10 mM MnCl_2_, and the error is the error of the fit. *B*, AlphaFold2 structure of SpoIIAA showing the SpoIIE and SpoIIAB binding interface outline. The SpoIIAA structure on the *left* shows the phosphorylation site, S58 (*red* and *light pink*), and the residue R67 (*blue* and *light blue*). The SpoIIE binding interface is outlined (*red*) based on a 1.4 Å probe radius, overlapping both S58 and R67. SpoIIE residue E675 (*blue*) is in proximity to R67. On the *right*, the SpoIIAB/SpoIIAA binding interface is outlined (*red*) based on a 1.4 Å probe radius. Phosphorylation site S58 (*pink*) and R67 (*blue* and *light blue*) are notated on the structure.

To determine how features of the substrate protein interact with specificity determinants in the phosphatase domain, we generated a variant of SpoIIAA with arginine 67 substituted with threonine (the homologous residue of RsbV). We found that SpoIIAA^R67T^ was dephosphorylated roughly 20-fold slower by SpoIIE (measured under k_cat_/K_M_ conditions) (Fig. 5*A*). Remarkably, R67 of SpoIIAA is predicted to be in proximity of E675 of SpoIIE in our AlphaFold2 model of the SpoIIAA/SpoIIE complex (Fig. 5*B*). We therefore assayed dephosphorylation of SpoIIAA^R67T^ by SpoIIE^E675S^ to determine if the substitutions were compensatory. Indeed, we found that the activity of SpoIIE^E675S^ was greater toward SpoIIAA^R67T^ than SpoIIAA (Fig. 5*A*). Importantly, this was not the case for SpoIIE^I723S^, for which we observed a 2-fold reduction in rate regardless of the substrate (Fig. S6). This finding provides further support for the AlphaFold2 model of the SpoIIE/SpoIIAA complex and emphasizes the importance of the loop connecting α2 (part of the switch element) to β4 of the PPM fold for specificity. The position of R67 on SpoIIAA additionally demonstrates that docking interactions involving the three-dimensional structure, distant from the phosphorylation site of the substrate protein, are critical for recognition by the phosphatase.

### Opposing kinases and phosphatases recognize distinct substrate features

One important aspect of phosphatase substrates is that they must first be recognized and phosphorylated by a kinase. We therefore compared the docking interface of SpoIIAA with SpoIIE and with its cognate kinase, SpoIIAB (for which a cocrystal structure has been determined) (21). The interfaces were substantially nonoverlapping, with much more extensive contacts formed between SpoIIAB and SpoIIAA than SpoIIE and SpoIIAA (Fig. 5*B*). This divergence of substrate interaction interface between kinase and phosphatase suggests that substrate specificity has the potential to evolve independently for each enzyme. However, R67 was part of both interfaces, suggesting some overlap in key specificity determinants.

## Discussion

To control critical transcriptional programs, SpoIIE and RsbU must discriminate between their paralogous substrate proteins with high fidelity. How this specificity is achieved was a mystery because both phosphatases present their active sites on the solvent-exposed surface and have similar K_M_ for both substrates (8). Here, we discovered that SpoIIE and RsbU have three variable loops that recognize the cognate substrate protein and facilitate dephosphorylation through a conserved allosteric element. Because related phosphatase/substrate pairs control diverse transcriptional programs across bacterial species, we postulate that this mechanism is generalizable, providing a molecular framework for understanding phosphatase specificity. Below we discuss the implications of this mechanism for the evolution of new signaling pathways and for the broader PPM family of phosphatases.

### Substrate docking interactions control phosphatase activity

We discovered that there are three variable loops on the PPM phosphatase domain that discriminate between substrates through direct interactions: the switch-loop (between α2 and β4), the flap (between β7 and β8), and the α3/4 loop. Substitutions of single amino acids in each loop were sufficient to change substrate preference by as much as 10-fold, and combining three substitutions was sufficient for 500-fold change in specificity. Intriguingly, the effect of these substitutions on specificity was entirely through decreasing the k_cat_ for the cognate substrate and increasing the k_cat_ for the noncognate substrate. We therefore infer that these docking interactions are critical for positioning the substrate protein and organizing the active site. Future high-resolution experimental structures will be required to reveal the specific structural changes that are required for phosphatase activation. However, we discovered a second class of substitutions that made SpoIIE and RsbU more promiscuous and are buried in the core of the phosphatase domain, interacting with a conserved switch element that controls metal cofactor binding and catalysis. Importantly, the flap and switch-loop directly contact the switch element, suggesting a mechanism for how substrate docking is transduced to control the active site.

### Conservation of substrate docking interactions

Our analysis of PPM phosphatase domain sequences confirmed that the switch-loop, flap, and the α3/4 loop are variable regions across PPM family phosphatases, supporting the possibility that they may be conserved elements for achieving substrate specificity. There is one structure in the PDB of a phosphatase bound to its substrate protein, the *A. thaliana* drought-tolerance response phosphatase, HAB1, bound to its substrate, the kinase SnRK2.6 (28) (Fig. S8). This structure revealed that W385 of the HAB1 flap is a critical latch for interaction with both SnRK2.6 and the competing abscisic acid receptor PYR1. Similar to SpoIIE^I723^ and RsbU^S251^, they additionally identified flap residues V393 and Y404 that are more proximal to the switch element of HAB1 as making important contacts to SnRK2.6. Our analysis of the structure additionally reveals that HAB1^E323,T324^ in the switch-loop (at the equivalent position of SpoIIE^E675^ and RsbU^S251^) and HAB1^S490^ in the α3/4 loop (at the equivalent position of SpoIIE^P752^ and RsbU^R280^) make contacts to SnRK2.6 but not PYR1. This suggests that these critical features for substrate recognition are conserved across kingdoms despite the evolutionary divergence between the phosphatases and despite the fact that they act on unrelated substrate proteins. Supporting this conclusion, the flap region has also been shown to contribute to the specificity of human PPM phosphatases (24, 25, 26).

### The evolutionary diversification of phosphatases

The canonical evolutionary model for how proteins evolve new substrate specificity is that following a gene duplication event, mutations create a promiscuous intermediate before subsequent mutations block recognition of the original substrate and optimize recognition of the new substrate (29). Our findings suggest that PPM phosphatases have a built-in path for this evolution, with mutations in the switch element causing substrate promiscuity and variable loop regions directly interacting with substrates providing discrimination. We speculate that this ordered pathway may underly the diversification of PPM phosphatases, particularly in species of bacteria and plants that have large numbers of PPM phosphatases (often more than 50) (7, 30).

The evolution of phosphatase specificity is additionally constrained by the fact that each substrate protein is necessarily also the substrate of an opposing protein kinase. For SpoIIAA, the phosphatase/substrate and kinase/substrate interfaces are largely nonoverlapping, suggesting that there is significant room for phosphatases and kinases to independently evolve specificity for the same substrate proteins. Whether these same principles hold for other kinase/phosphatase pairs will be of significant interest as the mechanism of substrate discrimination is uncovered for more phosphatases.

## Experimental procedures

### Strain construction

Strains were grown in liquid Lennox lysogeny broth (LB, Sigma Aldrich) or on plates supplemented with 15% Bacto agar (Difco). Competence medium was used during *Bacillus* transformation. Antibiotics were added where appropriate to select for plasmids and for transformant selection, but strains with genomic markers were not generally grown on selective media. Isopropyl-beta-D-thiogalactoside (IPTG) was used at 1 mM, and 5-bromo-4-chloro-3-indolyl-beta-D-galacto-pyranoside (X-gal) was used at 80 μg/ml. The antibiotics used were macrolide-lincosamide-streptogramin (MLS; 0.5 μg/ml erythromycin, 2.5 μg/ml lincomycin), tetracycline (25 μg/ml), chloramphenicol (20 μg/ml for *E. coli*), carbenicillin (100 μg/ml), zeomycin (comparable to phleomycin 0.4 μg/ml), spectinomycin (100 μg/ml), and kanamycin (10 μg/ml for *B. subtilis* or 50 μg/ml for *E. coli*). Standard molecular biology techniques were used to construct DNA plasmids using isothermal assembly (Gibson cloning) to generate new constructs and Quikchange mutagenesis for site-directed mutagenesis. Table S1 provides all strains, and Table S2 provides all primers used in this study.

All strains were constructed in the *B. subtilis* PY79 strain background. To make the σ^F^ reporter strain, genomic DNA from *B. subtilis* strains containing *ΔrsbR rsbS rsbT rsbU rsbV rsbW sigB rsbX::kan* (19), *amyE::pspoIIQ-lacZ cm* (31), and *spoIIAB::spoIIAB*^*R105C*^ *spec* (27) genetic modifications were sequentially transformed into the *ΔspoIIE::phleo* (27) parent strain. Transformants were selected based on antibiotic resistance and the ability to break down starch. The *spoIIAB*^*R105C*^ *spec* construct was introduced by long flanking homology PCR, and its sequence was confirmed by colony PCR with primers flanking the spoIIAA operon for sequencing. The σ^F^ expressing strain for testing viability during vegetative growth was generated by sequentially transforming a *Bacillus subtills* strain with *ywrk::Tn917::amyE::pspank-spoIIAoperon tet* (32) with genomic DNA containing *ΔrsbR rsbS rsbT rsbU rsbV rsbW sigB rsbX::kan*, *amyE::pSpoIIQ-lacZ cm*, and *ΔspoIIE::phleo*. The σ^B^ reporter strains were constructed as described previously (8).

### *σ*^F^ toxicity assay

Strains were grown in LB/MLS to an *A*600 of approximately 0.35. Cells were 10-fold serially diluted and spotted on LB, MLS, and X-gal, with and without IPTG at 37 °C overnight. Another aliquot of the culture was plated on LB/MLS, and a Whatman paper disc approximately 1 cm that was saturated with 50 μl of 1M IPTG was added to the center of the plate after spreading the cells. The cells were grown at 37 °C overnight.

### Genetic screen

*B. subtilis* genomic DNA containing RsbT/RsbU was amplified by PCR using GoTaq DNA polymerase mix for 30 cycles without modification from the manufacturers protocol (Promega). DNA sequencing revealed that inserts had on average one to two mutations per product. Pools of the mutagenized PCR product were assembled into the pHB201 digested vector using isothermal (Gibson) assembly and transformed into *E. coli* DH5α cells. *E. coli* cells were pooled, and plasmid DNA was extracted and transformed into the *Bacillus* screen strain using natural competence. *B. subtilis* cells were plated on Difco sporulation medium containing MLS, IPTG, and X-gal. Blue colonies were selected and restruck on Difco sporulation medium plates with and without IPTG, as well as LB/MLS plates. Plasmids from colonies that retested as σ^F^ positive were isolated and inserts were sequenced by Sanger sequencing.

### Protein expression and purification

All proteins were expressed in *E. coli BL21 (DE3)* cells grown at 37 °C to an *A*600 of 0.4 and induced at 16 °C for 14 to 18 h with 1 mM (IPTG). Cells were harvested and purified as follows (protocols adapted with modification from (6, 8, 9)).

#### SpoIIE and SpoIIE variants

Cell pellets were resuspended in lysis buffer with 1 mM phenylmethylsulfonyl fluoride (PMSF) [50 mM K•Hepes, pH 8, 200 mM NaCl, 20 mM imidazole, 10% glycerol, 0.5 mM dithiothreitol (DTT)] and were lysed using three passes in a microfluidizer at 10,000 PSI. Cell lysates were cleared by spinning at 16,000 RPM for 30 to 45 min in an Avanti JA-20 rotor. Cleared lysates were then bound to Ni-NTA resin (2 ml/L of culture) on the column by gravity flow after the Ni-NTA resin slurry was equilibrated with lysis buffer. The resin was then washed with 10 CV of lysis buffer containing 20 mM imidazole and eluted with 200 mM imidazole. The 6-His tag was cleaved with 3C protease in dialysis with lysis buffer at 4 °C overnight. The 6-His cleaved tag and 3C protease was subtracted by passing over the Ni-NTA resin. The protein was spin-concentrated before the gel filtration run. It was further purified on a Superdex 75 16/60 column that was equilibrated in 20 mM K•Hepes, pH 8, 50 mM NaCl, 2 mM DTT on the AKTA FPLC. The fractions were pooled, concentrated approximately between 46 and 200 μM, flash-frozen, and stored at −80 °C.

#### SpoIIAA and SpoIIAA variants

Cell pellets were resuspended in lysis buffer with 1 mM PMSF (50 mM K•Hepes, pH 8, 100 mM NaCl, 20 mM imidazole, 10% glycerol, and 0.5 mM DTT) and were lysed using two passes in a microfluidizer at 10,000 PSI. Cell lysates were cleared by spinning at 16,000 RPM for 30 min in a Sorvall SS-34 rotor. Cleared lysates were run over a HisTrap HP column on an AKTA FPLC. Fractions were pooled, and the 6-His tag was cleaved with 3C protease in dialysis with lysis buffer at 4 °C overnight. The 6-His cleaved tag and 3C protease were subtracted by passing over equilibrated Ni-NTA resin. Protein was spin-concentrated before the gel filtration run. It was further purified on a Superdex 75 16/60 column that was equilibrated in 50 mM K•Hepes, pH 8, 100 mM NaCl, 10% glycerol, and 2 mM DTT on the AKTA FPLC. The fractions were pooled, concentrated approximately between 270 and 500 μM, flash-frozen, and stored at −80 °C.

#### SpoIIAA-P

Cell pellet was resuspended in lysis buffer with 1 mM PMSF (50 mM K•Hepes, pH8, 100 mM NaCl, 20 mM imidazole, 10% glycerol, 0.5 mM DTT) and was lysed using two passes on a microfluidizer at 10,000 PSI. Cell lysate was cleared by spinning at 16,000 RPM for 30 min in an Avanti JA-20 rotor. Cleared lysate was then bound to Ni-NTA resin (2 ml/L of culture) on the column by gravity flow after the Ni-NTA resin slurry was equilibrated with lysis buffer. The resin was then washed with 10 CV of lysis buffer containing 20 mM imidazole and eluted with 200 mM imidazole. The 6-His tag was cleaved with 3C protease in dialysis with lysis buffer at 4 °C overnight. The 6-His cleaved tag and 3C protease was subtracted by passing over the Ni-NTA resin. The protein was spin-concentrated before the gel filtration run. It was further purified on a Superdex 200 column that was equilibrated in 50 mM K•Hepes, pH 8, 100 mM NaCl, 10% glycerol, and 2 mM DTT. The fractions were pooled, concentrated approximately 200 to 300 μM, flash-frozen, and stored at −80 °C.

#### RsbV

BL21 (DE3) cells were transformed with pET47bRsbV each time before the protein was expressed. Cell pellets were resuspended in lysis buffer with 200uM PMSF (50 mM K•Hepes, pH 8, 200 mM NaCl, 20 mM imidazole, 10% glycerol, 0.5 mM DTT) and were lysed using two passes on a microfluidizer at 10,000 PSI. Cell lysate was cleared by spinning at 16,000 RPM for 45 min in an Avanti JA-20 rotor. Cleared lysates were then bound to Ni-NTA resin (2 ml/L of culture) on the column by gravity flow after the Ni-NTA resin slurry was equilibrated with lysis buffer. The resin was then washed with 10 CV of lysis buffer containing 20 mM imidazole and eluted with 200 mM imidazole. The 6-His tag was cleaved with 3C protease in dialysis with lysis buffer at 4 °C overnight. The 6-His cleaved tag and 3C protease was subtracted by passing over the Ni-NTA resin. The protein was spin-concentrated before the gel filtration run. It was further purified on a Superdex 75 16/60 column that was equilibrated in 50 mM K•Hepes, pH 8, 100 mM NaCl, 10% glycerol, and 2 mM DTT. The fractions were pooled, concentrated approximately 150 μM, flash-frozen, and stored at −80 °C.

#### SpoIIAB

Cell pellets were resuspended in lysis buffer with 1 mM PMSF (50 mM K•Hepes, pH 7.5, 200 mM NaCl, 10 mM MgCl_2_, 20 mM imidazole, 10% glycerol, and 0.5 mM DTT) and were lysed using two passes in a microfluidizer at 10,000 PSI. Cell lysates were cleared by spinning at 16,000 RPM for 30 min in a Sorvall SS-34 rotor. Cleared lysates were run over a HisTrap HP column on an AKTA FPLC. Fractions were pooled, and the protein was spin-concentrated before the gel filtration run. It was further purified on a Superdex 75 16/60 column that was equilibrated in 50 mM K•Hepes, pH 7.5, 175 mM NaCl, 10% glycerol, 10 mM MgCl2, and 1 mM DTT on the AKTA FPLC. The 6H-sumo tag was left uncleaved to aid with removal during phosphorylation reactions. The fractions were pooled, concentrated to approximately 200 μM, flash-frozen, and stored at −80 °C.

### Phosphatase assays

Phosphatase assays were performed with SpoIIAA that was labeled with ^32^P by incubating SpoIIAA (45 μM), 6His-sumo-SpoIIAB (55 μM), and 50 μCi of γ-^32^P ATP overnight at room temperature in 50 mM K•Hepes, pH 7.5, 50 mM KCl, 2 mM DTT, and 0.75 mM MgCl_2_. Unincorporated nucleotide was removed by buffer exchange using a Zeba spin column (Pierce) equilibrated with 25 mM K•Hepes, pH 7.5, 200 mM NaCl. 6H-sumo-SpoIIAB was removed by incubating it with Q-Sepharose resin equilibrated in 50 mM K•Hepes, pH 7.5, 50 mM KCl, 2 mM DTT, and 0.75 mM MgCl_2_. The flowthrough fraction from the Q Sepharose resin containing SpoIIAA-^32^P was then exchanged into 50 mM K•Hepes, pH 8, 100 mM NaCl using a Zeba spin column to remove any unincorporated nucleotide and free phosphate. The labeled SpoIIAA-^32^P was aliquoted and frozen at −80 °C for future use.

To produce ^32^P-labeled RsbV-P, 40 μM RsbV, 45 μM 6His-RsbW, and 100 μCi of γ-^32^P ATP were incubated overnight at room temperature in 50 mM K•Hepes, pH7.5, 50 mM KCl, 10 mM MgCl2, and 2 mM DTT. Unincorporated nucleotide was removed by buffer exchange using Zeba column equilibrated in 50 mM K•Hepes, pH 8, 100 mM NaCl. 6His-RsbW was then removed by Ni-NTA resin equilibrated in 50 mM K•Hepes, pH 8, 100 mM NaCl, and 20 mM imidazole. The RsbV-^32^P flow-through fraction from the Ni-NTA resin was then buffer exchanged into 50 mM K•Hepes, pH 8, 100 mM NaCl buffer using three sequential Zeba spin columns to remove all unincorporated nucleotide and free phosphate. The labeled RsbV-^32^P was aliquoted and frozen at −80 °C.

All phosphatase assays were conducted at room temperature in 50 mM K•Hepes, pH8, and 100 mM NaCl. The concentrations of enzyme, substrate, and metal cofactor (MnCl_2_ or MgCl_2_) were varied as indicated. SpoIIAA reactions had 0.2 mg BSA added to prevent protein adhesion to reaction tubes. Reactions were stopped with 0.5 M EDTA, pH 8, and 2% SDS, then run on PEI-Cellulose TLC plates that were developed in 1 M LiCl2 and 0.8 M acetic acid. The plates were imaged on an Amersham typhoon scanner and quantified with ImageQuant. Each experiment was independently replicated to determine the appropriate timepoints and concentrations used for the final experiment shown in the figure. The error reported is from the error of the fit for a final experiment with optimized timepoints.

### p-Nitrophenyl phosphate

p-Nitrophenyl phosphate assay was conducted at room temperature by mixing 50 mM Hepes, pH 8, 100 mM NaCl, 0.5 uM of enzyme, 20 mM MnCl2, and increasing concentrations of PNPP (0.5 mM-25 mM) in a 96-well plate. Reactions were started with PNPP, and hydrolysis of PNPP to p-nitrophenol was measured at 405 nm in a plate reader.

### AlphaFold2 structure predictions

AlphaFold2 predictions were performed using ColabFold (33). Alphafold2_multimer_v2 was used in unpaired_paired mode with no templates with three recycles, 200 iterations, and greedy pairing strategy. The predicted aligned error plots for all AlphaFold2 structures are shown in Fig. S7.

## Data availability

All data are contained within the manuscript.

## Supporting information

This article contains supporting information.

## Conflict of interest

The authors declare no conflict of interest with the contents of this article.
