# Supplementary material for: Docking interactions determine substrate specificity of members of a widespread family of protein phosphatases
Source: J Biol Chem. 2024 Aug 22;300(9):107700. doi: 10.1016/j.jbc.2024.107700 (PMC11418112; doi:10.1016/j.jbc.2024.107700)
Supplement: Supporting Figures [file mmc1.pdf]

Supplemental Figure 1

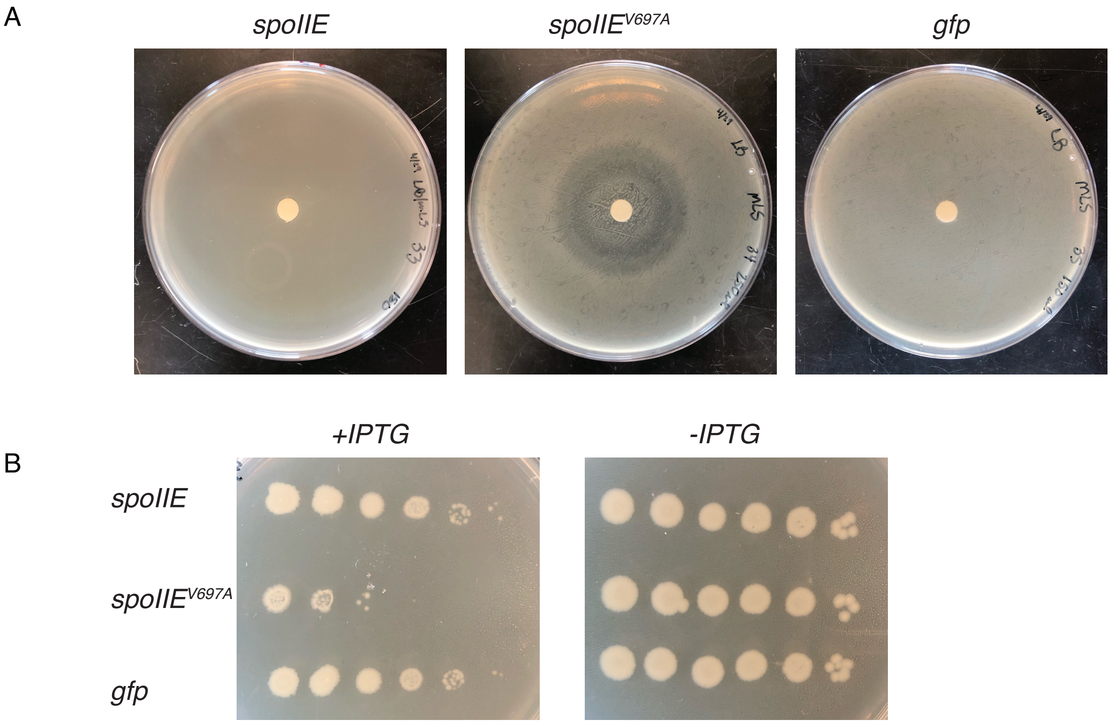

Supplemental Figure 1: Activation of σ^F during vegetative growth is toxic to cells

A. Strains with σ^F expression under nonsporulating conditions with a *lacZ* reporter for σ^F activity. Strain expressing either *spoIIE* (left plate) *spoIIE*^{V697A} (middle plate) or *gfp* (right plate) from plasmid pHB201 were grown on LB/MLS plates. A Whatman paper disk of approximately 1cm was saturated with 1M IPTG and placed in the center of the plate. Plates were incubated at 37°C overnight. **B.** Strain expressing either *spoIIE* (top) *spoIIE*^{V697A} (middle) or *gfp* (bottom) from plasmid pHB201 were serial diluted 10-fold and grown on LB/MLS plates, plus (left) and minus (right) 1 mM IPTG.

Supplemental Figure 2

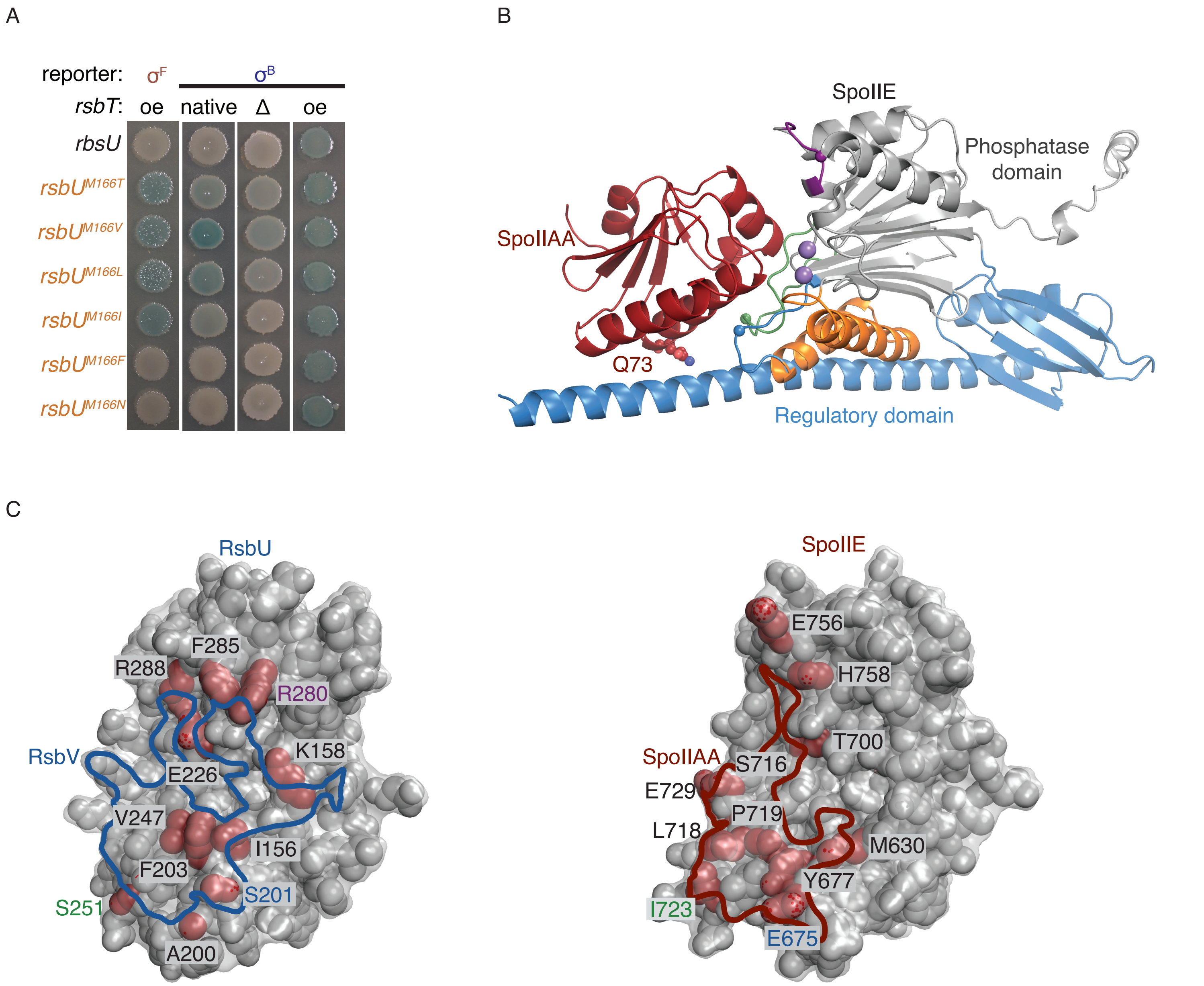

Supplemental Figure 2: Conservation of phosphatase/substrate recognition

A. Strains carrying plasmid expressing M166X mutation located in the switch of RsbU. Reporter strains have *lacZ* under the control of either σ^F (left row) or σ^F (last three rows) promoter. *rsbT* is overexpressed in the σ^F reporter strain. *rsbT* in the σ^B reporter strains is either expressed from the native locus (second column from left), deleted (third column from left), or overexpressed (last column). M166X mutation series are M166T, M166V, M166L, M166I, M166F, and M166N. **B.** AlphaFold2 Structure of *B. subtilis* SpoIIE monomer and SpoIIAA showing contact interface between the two proteins. The phosphatase domain of SpoIIE (gray), along with the elements located in the phosphatase domain, switch (orange), flap (green), 2/4 loop (blue), and the 3/4 loop, are noted on the structure. The metal ions (purple) are visible in the active site. Residues E675S (blue), and I723 (green), are represented by spheres within the α1/β4 loop and flap, respectively. Position of the SpoIIE regulatory domain (blue) in relation to SpoIIAA (red). SpoIIAA shows the position of residue Q73 (red and blue spheres) in relation to the regulatory domain of SpoIIE. **B.** AlphaFold2 structure of *B. subtilis* RsbU (left) and SpoIIE (right). The phosphatase domain (gray) with non-conserved residues (red) whose side chains contact the side chains of RsbV and SpoIIAA, respectively. The RsbU/RsbV binding interface is outlined in blue, and SpoIIE/SpoIIAA in red based on 1.4 Å probe radius.

Supplemental Figure 3

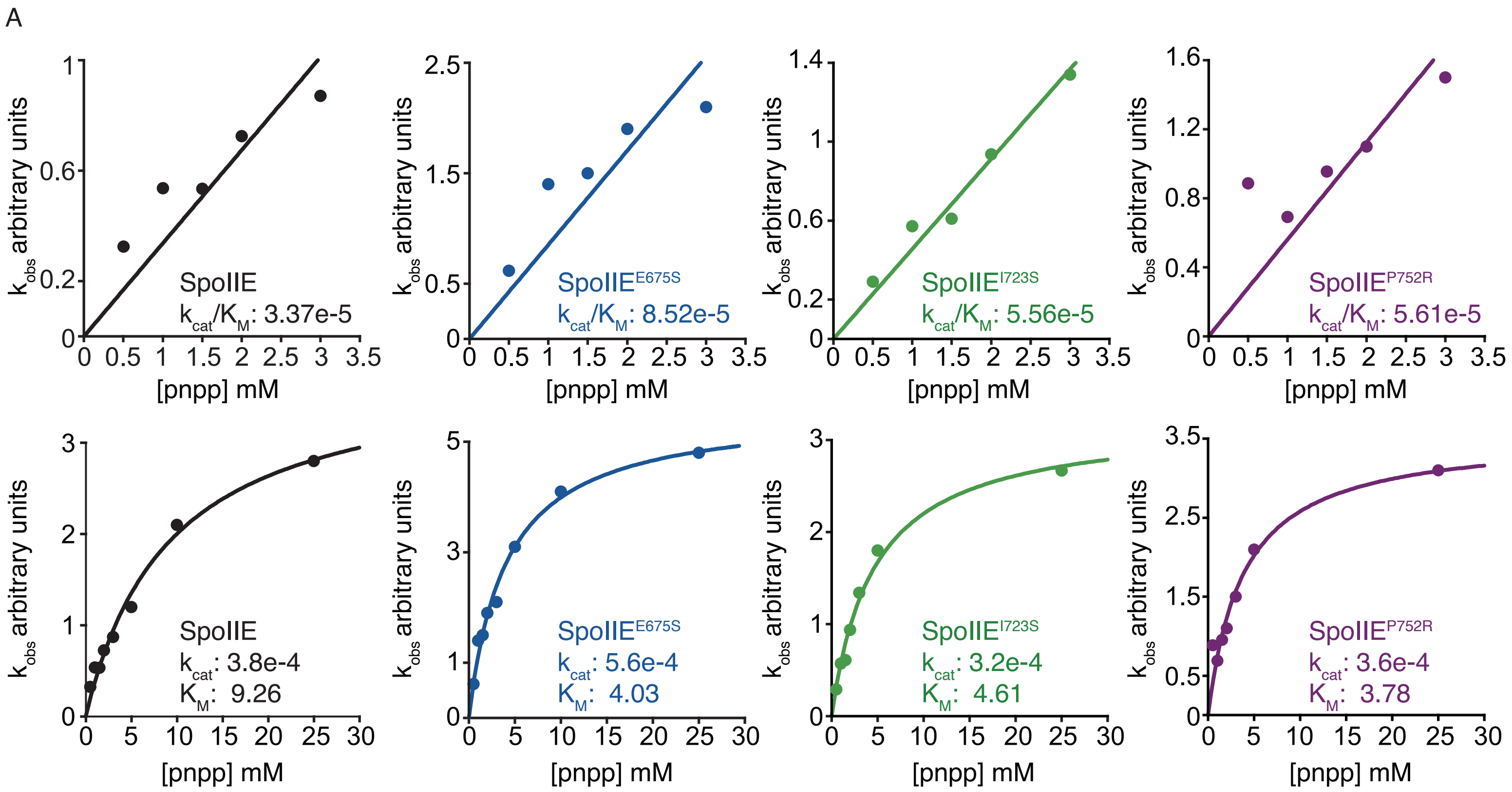

Supplemental Figure 3: Changes in phosphatase activity are substrate-specific

A. The rate of p-nitrophenyl phosphate dephosphorylation during the initial linear phase was measured for SpoIIE, SpoIIE^{E675S}, SpoIIE^{I723S}, and SpoIIE^{P752R}. Observed velocities (in arbitrary absorbance units per time) were plotted as a function of pnpp concentration. Top plots were fit to the linear equation (in KaleidaGraph) (k_{cat}/K_M)*[pnpp]. The lower plots were fit to the Michaelis-Menten equation (in KaleidaGraph) k_{cat}*[pnpp]/(K_M + [pnpp]).

A

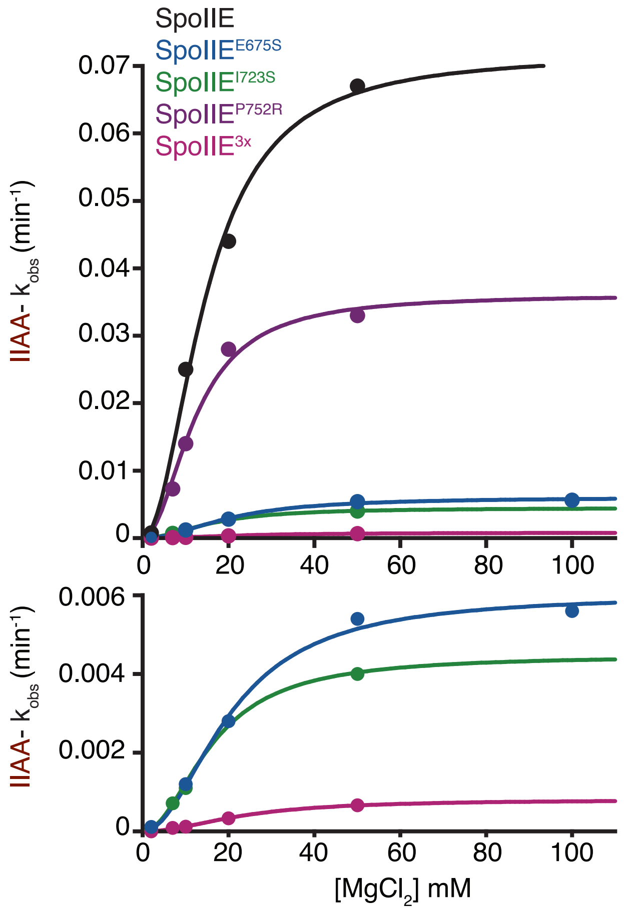

B

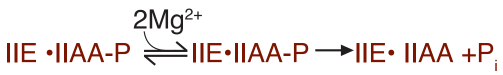

	K_M (mM)	k_{obs} (min^{-1})
SpoIIE	14.7	0.072
SpoIIE^{E675S}	20.5	0.0060
SpoIIE^{I723S}	16.3	0.0045
SpoIIE^{P752R}	12.4	0.036
SpoIIE^{3x}	23.6	0.00080

Supplemental Figure 4: Recruitment of metal cofactor is not impacted

A. Activity of SpoIIE (black), SpoIIE^{E675S} (blue), SpoIIE^{I723S} (green), SpoIIE^{P752R} (purple), and SpoIIE^{3x} (triple mutation of E675S, I723S, and P752R, pink) as a function of $MgCl_2$ concentration with SpoIIAA. The data plotted was fit to a cooperative model using the equation $k_{obs}=k_{cat}*[MgCl_2]^2/(K_M^2+[MgCl_2]^2)$. The measured K_M were SpoIIE 14.7± 1.2 μM, SpoIIE^{E675S} 20.5± 1.4 μM, SpoIIE^{I723S} 16.3± 0.81 μM, SpoIIE^{P752R} 12.4± 1.0 μM and SpoIIE^{3x} 23.6± 1.2 μM. The error is the error of the fit. The reactions were single turnover reactions with varying concentrations of $MgCl_2$, 1μM SpoIIE, and 0.05 SpoIIAA. **B.** Graphic scheme showing a summary of the dephosphorylation reaction of SpoIIAA by SpoIIE.

Supplemental Figure 5

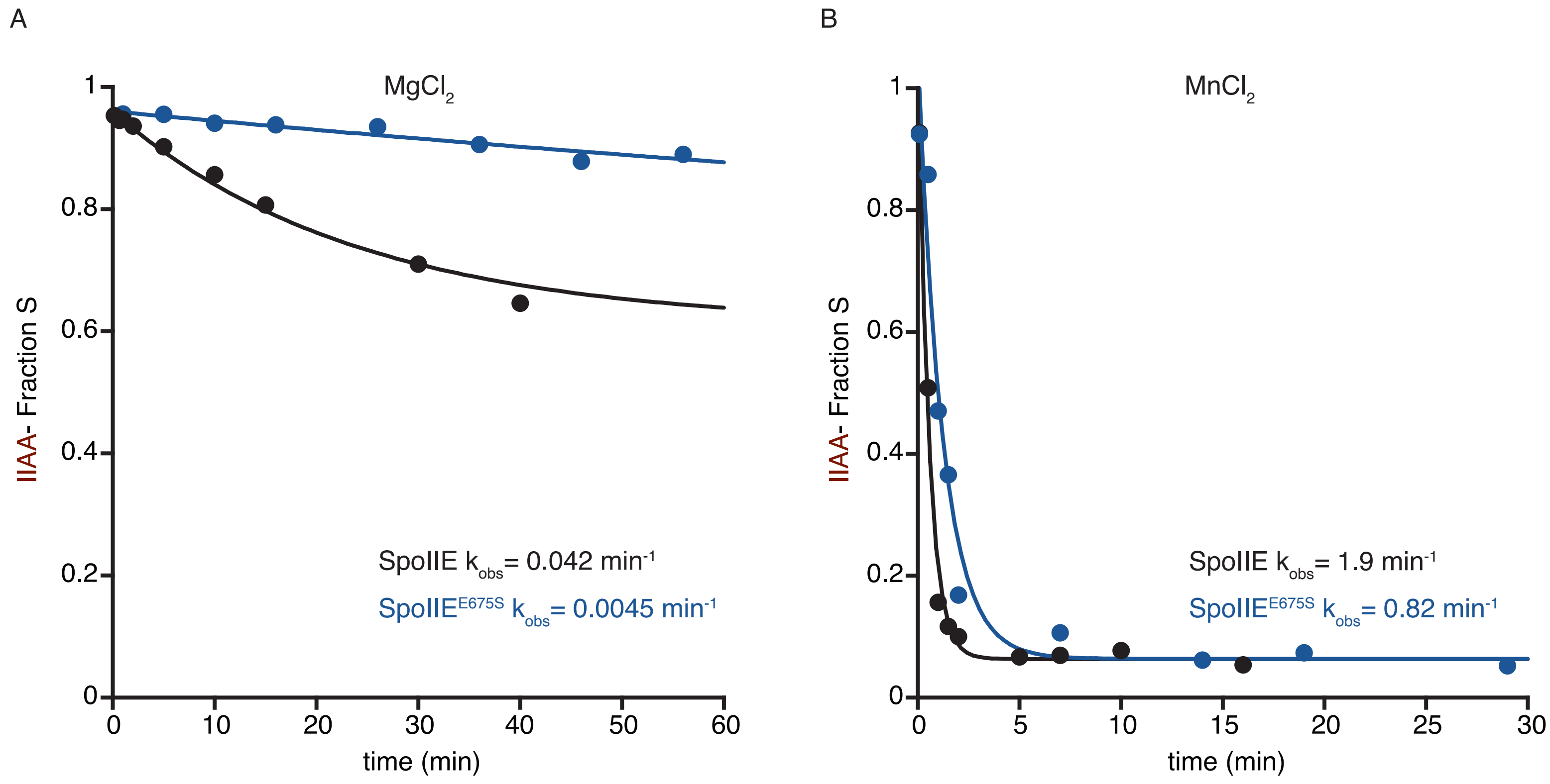

Supplementary Figure 5: Metal cofactor influences catalysis of cognate substrate

Activity of SpoIIE (black) and SpoIIE^{E675S} (blue) with SpoIIAA with either $MgCl_2$ or $MnCl_2$. Reactions measured the fraction of SpoIIAA as it decreased over time. **A**. Single turnover reactions were conducted with 1 µM SpoIIE, 0.5 µM SpoIIAA-P, and 10 mM $MgCl_2$. The k_{obs} were SpoIIE 0.042± 0.0023 min^{-1} and SpoIIE^{E675S} 0.0045± 0.00036 min^{-1}. The data was fit to an exponential decay function. The error is the error of the fit. **B.** Single turnover reactions were conducted with 1 µM SpoIIE, 0.5 µM SpoIIAA-P, and 10 mM $MnCl_2$. The k_{obs} were SpoIIE 1.9± 0.18 min^{-1} and SpoIIE^{E675S} 0.82± 0.14 min^{-1}. The data was fit to an exponential decay function. The error is the error of the fit.

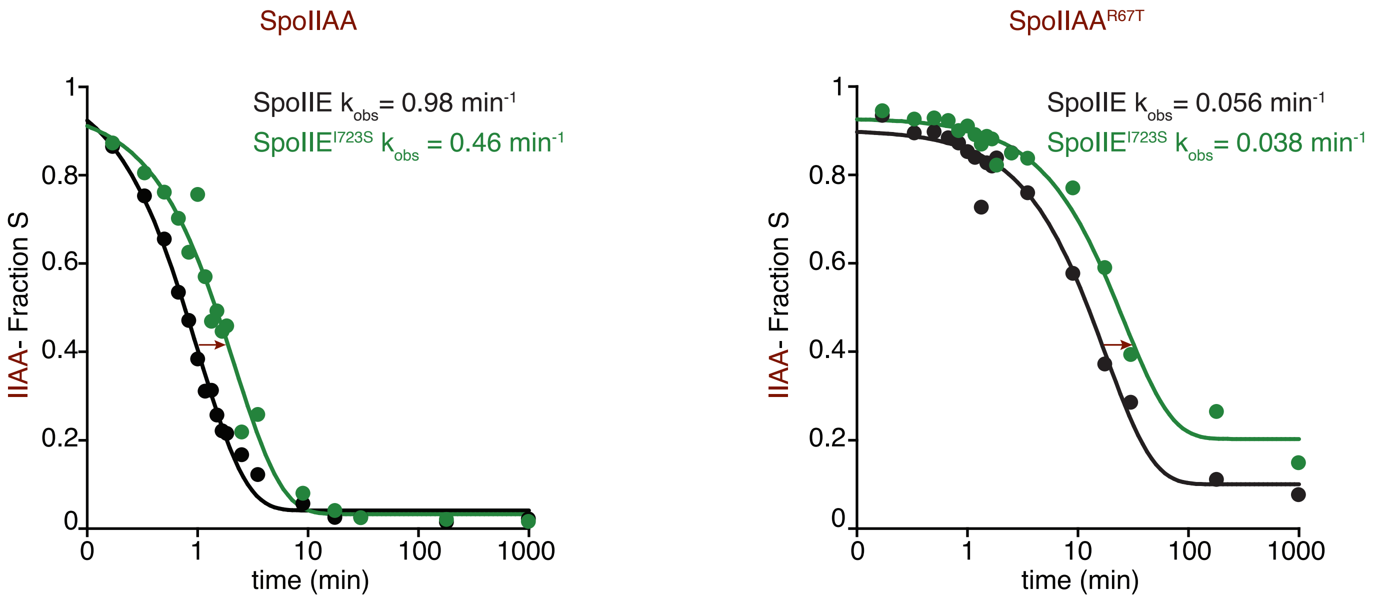

Supplemental Figure 6: SpoIIE^{I723S} does not complement SpoIIAA^{R67T}

A. Rate of SpoIIAA-P and SpoIIAA^{R67T} dephosphorylation by SpoIIE and SpoIIE^{I723S} over time. The plot on the *left* measures the fraction of SpoIIAA-P dephosphorylated over time by SpoIIE (black) and SpoIIE^{I723S} (green) and was fit to an exponential decay function. The measured k_{obs} were SpoIIE 0.98± 0.05 min^{-1} and SpoIIE^{I723S} 0.46± 0.049 min^{-1}. The *right* plot measures the fraction of SpoIIAA^{R67T} dephosphorylated over time by SpoIIE (gray) and SpoIIE^{I723S} (light green) and was fit to an exponential decay function. The k_{obs} were SpoIIE 0.056± 0.0061 min^{-1} and SpoIIE^{I723S} 0.038± 0.0039 min^{-1}. Reactions were single-turnover reactions using 0.1 µM SpoIIE, 0.5 µM SpoIIAA-P, 10 mM $MnCl_2$, and the error is the error of the fit.

Supplemental Figure 7

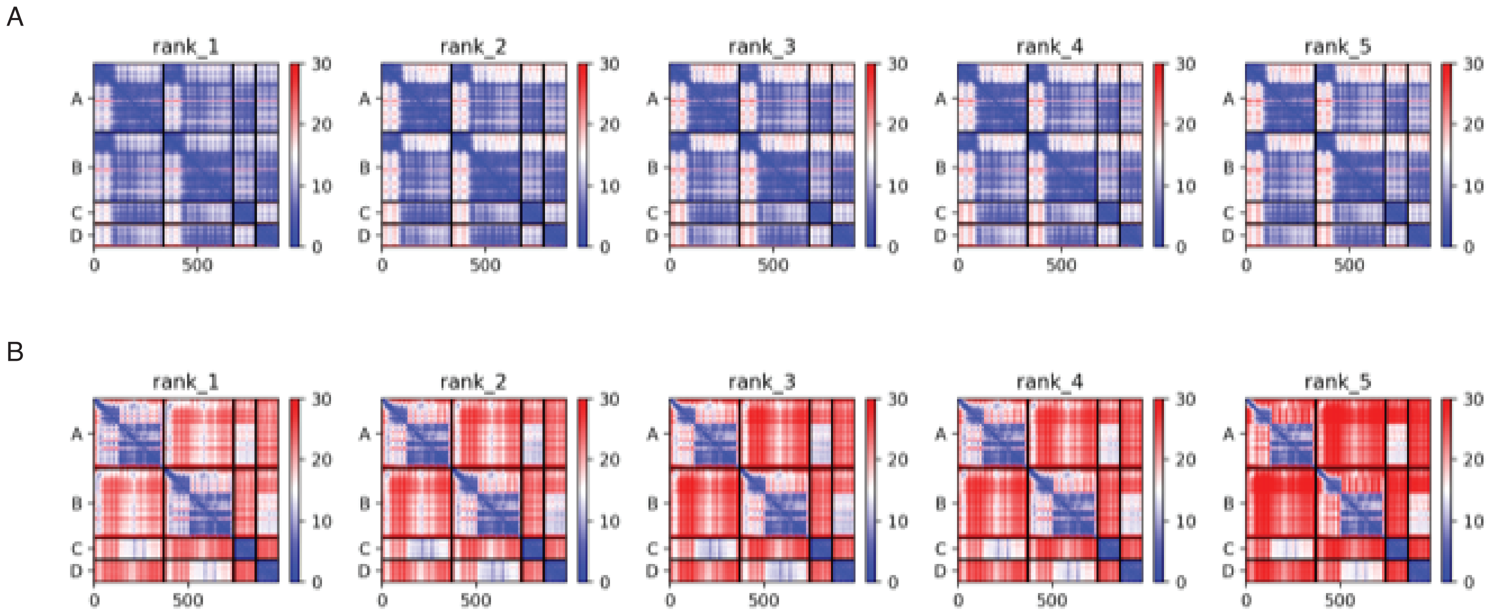

Supplemental Figure 7: PAE plots of AlphaFold2 structure predictions

Predicted aligned error of the AlphaFold2 structure prediction of the following proteins: **A.** *B. subtilis* RsbU (chain A and B), and RsbV (chain C and D **B.** *B. subtilis* SpoIIE (chain A and B) and SpoIIAA (chain C and D).

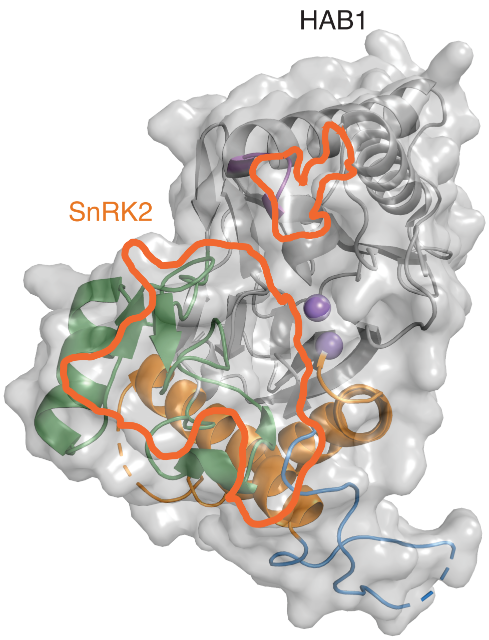

Supplemental Figure 8: HAB1/SnRK2 interface show contact with flap, switch and α2/β4 loop

Crystal structure of *Arabidopsis thaliana* HAB1 depicting binding interface with SnRK2 outlined in orange based on a 1.4 Å probe radius. Interface outline shows SnRK2 makes contact with flap (green), switch (orange) and α2/β4 loop (blue).
