# Supplementary material for: Docking interactions determine substrate specificity of members of a widespread family of protein phosphatases
Source: J Biol Chem. 2024 Aug 22;300(9):107700. doi: 10.1016/j.jbc.2024.107700 (PMC11418112; doi:10.1016/j.jbc.2024.107700)
Supplement: Supporting Tables [file mmc2.pdf]

Supplementary Table 1:

B. subtilis strains (PY79 background)

Strain #	Genotype	Reference
SP32	*Ywrk::amyE::pspank-spoIIAoperon tet, ΔspoIIE::phleo, amyE::pspoIIQ-lacZ cm,Δ(rsbR rsbS rsbT rsbU rsbV rsbW sigB rsbX)::kan*	This paper
SP33	*Ywrk::amyE::pspank-spoIIAoperon tet, ΔspoIIE::phleo, amyE::pspoIIQ-lacZ cm, Δ(rsbR rsbS rsbT rsbU rsbV rsbW sigB rsbX)::kan, pHB201-P(spank)- WT rsbT rsbU*	This paper
SP34	*Ywrk::amyE::pspank-spoIIAoperon tet , ΔspoIIE::phleo , amyE::pspoIIQ-lacZ cm, Δ(rsbR rsbS rsbT rsbU rsbV rsbW sigB rsbX)::kan, pHB201-P(spank)-spoIIEV697A*	This paper
SP35	*Ywrk::amyE::pspank-spoIIAoperon tet , ΔspoIIE::phleo , amyE::pSpoIIQ-lacZ cm, Δ(rsbR rsbS rsbT rsbU rsbV rsbW sigB rsbX)::kan , pHB201-P(spank)-GFP*	This paper
SP49	*ΔspoIIE::phleo, Δ(rsbR rsbS rsbT rsbU rsbV rsbW sigB rsbX)::kan, ΔamyE::pspoIIQ-lacZ cm, ΔspoIIAB::spoIIABR105C spec*	This paper
SP51	*ΔspoIIE::phleo, Δ(rsbR rsbS rsbT rsbU rsbV rsbW sigB rsbX)::kan, ΔamyE::pspoIIQ-lacZ cm, ΔspoIIAB::spoIIABR105C spec, pHB201-P(spank)-gfp*	This paper
SP52	*ΔspoIIE::phleo, Δ(rsbR rsbS rsbT rsbU rsbV rsbW sigB rsbX)::kan, ΔamyE::pspoIIQ-lacZ cm, ΔspoIIAB::spoIIABR105C spec, pHB201-P(spank)-WT rsbT rsbU*	This paper
SP54	*ΔspoIIE::phleo, Δ(rsbR rsbS rsbT rsbU rsbV rsbW sigB rsbX)::kan, ΔamyE::pspoIIQ-lacZ cm, ΔspoIIAB::spoIIABR105C spec, pHB201-P(spank)-rsbT rsbU S251L*	This paper
SP57	*ΔspoIIE::phleo, Δ(rsbR rsbS rsbT rsbU rsbV rsbW sigB rsbX)::kan, ΔamyE::pspoIIQ-lacZ cm, ΔspoIIAB::spoIIABR105C spec, pHB201-P(spank)-rsbT rsbU M134L*	This paper
SP58	*ΔspoIIE::phleo, Δ(rsbR rsbS rsbT rsbU rsbV rsbW sigB rsbX)::kan, ΔamyE::pspoIIQ-lacZ cm, ΔspoIIAB::spoIIABR105C spec, pHB201-P(spank)-rsbT rsbU S201G*	This paper
SP59	*ΔspoIIE::phleo, Δ(rsbR rsbS rsbT rsbU rsbV rsbW sigB rsbX)::kan, ΔamyE::pspoIIQ-lacZ cm, ΔspoIIAB::spoIIABR105C spec, pHB201-P(spank)-rsbT rsbU S167P*	This paper
SP61	*ΔspoIIE::phleo, Δ(rsbR rsbS rsbT rsbU rsbV rsbW sigB rsbX)::kan, ΔamyE::pspoIIQ-lacZ cm (NB591), ΔspoIIAB::spoIIABR105C spec, pHB201-P(spank)-rsbT rsbU M166T*	This paper
SP68	*ΔspoIIE::phleo, Δ(rsbR rsbS rsbT rsbU rsbV rsbW sigB rsbX)::kan, ΔamyE::pspoIIQ-lacZ cm , ΔspoIIAB::spoIIABR105C spec, pHB201-P(spank)-rsbT rsbU M166V*	This paper
SP69	*ΔspoIIE::phleo, Δ(rsbR rsbS rsbT rsbU rsbV rsbW sigB rsbX)::kan, ΔamyE::pspoIIQ-lacZ cm, ΔspoIIAB::spoIIABR105C spec, pHB201-P(spank)-rsbT rsbU M166L*	This paper

SP74	*ΔspoIIE::phleo, Δ(rsbR rsbS rsbT rsbU rsbV rsbW sigB rsbX)::kan, ΔamyE::pspoIIQ-lacZ cm, ΔspoIIAB::spoIIABR105C spec, pHB201-P(spank)-rsbT rsbU M166I*	This paper
SP75	*ΔspoIIE::phleo, Δ(rsbR rsbS rsbT rsbU rsbV rsbW sigB rsbX)::kan, ΔamyE::pspoIIQ-lacZ cm, ΔspoIIAB::spoIIABR105C spec, pHB201-P(spank)-rsbT rsbU M166N*	This paper
SP84	*ΔspoIIE::phleo, Δ(rsbR rsbS rsbT rsbU rsbV rsbW sigB rsbX)::kan, ΔamyE::pspoIIQ-lacZ cm, ΔspoIIAB::spoIIABR105C spec, pHB201-P(spank)-rsbT rsbU M166F*	This paper
KH11	*ΔrsbPQ ΔrsbTU rsbV-FLAG amyE::ctc-lacZ, pHB201-P(spank)-rsbTU*	Ho, & Bradshaw 2021
SP76	*ΔrsbPQ ΔrsbTU rsbV-FLAG amyE::ctc-lacZ, pHB201-P(spank)-rsbT rsbUM134L MLS*	This paper
SP77	*ΔrsbPQ ΔrsbTU rsbV-FLAG amyE::ctc-lacZ, pHB201-P(spank)-rsbT rsbU M166T MLS*	This paper
SP78	*ΔrsbPQ ΔrsbTU rsbV-FLAG amyE::ctc-lacZ, pHB201-P(spank)-rsbT rsbU S167P MLS*	This paper
SP79	*ΔrsbPQ ΔrsbTU rsbV-FLAG amyE::ctc-lacZ, pHB201-P(spank)-rsbT rsbU M166V MLS*	This paper
SP80	*ΔrsbPQ ΔrsbTU rsbV-FLAG amyE::ctc-lacZ, pHB201-P(spank)-rsbT rsbU M166L MLS*	This paper
SP81	*ΔrsbPQ ΔrsbTU rsbV-FLAG amyE::ctc-lacZ, pHB201-P(spank)-rsbT rsbU M166I MLS*	This paper
SP82	*ΔrsbPQ ΔrsbTU rsbV-FLAG amyE::ctc-lacZ, pHB201-P(spank)-rsbT rsbU M166N MLS*	This paper
KH411	*ΔrsbPQ ΔrsbTU rsbV-FLAG amyE::ctc-lacZ, pHB201-P(spank)-rsbT rsbU S201G MLS*	This paper
KH412	*ΔrsbPQ ΔrsbTU rsbV-FLAG amyE::ctc-lacZ, pHB201-P(spank)-rsbT rsbU S251L MLS*	This paper
KH373	*ΔrsbPQ ΔrsbTU rsbV-FLAG amyE::ctc-lacZ, pHB201-P(spank)-rsbT rsbU M166F MLS*	This paper
KH150	*ΔrsbPQ ΔrsbTU rsbV-FLAG amyE::ctc-lacZ, pHB201-P(spank)-rsbU MLS*	Ho, & Bradshaw 2021
KH154	*ΔrsbPQ ΔrsbTU rsbV-FLAG amyE::ctc-lacZ, pHB201-P(spank)- rsbU M166V MLS*	Ho, & Bradshaw 20212021
KH156	*ΔrsbPQ ΔrsbTU rsbV-FLAG amyE::ctc-lacZ, pHB201-P(spank)-rsbU M166L MLS*	Ho, & Bradshaw 2021
KH326	*ΔrsbPQ ΔrsbTU rsbV-FLAG amyE::ctc-lacZ, pHB201-P(spank)-rsbU M166I MLS*	This paper
KH327	*ΔrsbPQ ΔrsbTU rsbV-FLAG amyE::ctc-lacZ, pHB201-P(spank)-rsbU M166F MLS*	This paper
KH386	*ΔrsbPQ ΔrsbTU rsbV-FLAG amyE::ctc-lacZ, pHB201-P(spank)-rsbU M166T MLS*	This paper
KH387	*ΔrsbPQ ΔrsbTU rsbV-FLAG amyE::ctc-lacZ, pHB201-P(spank)-rsbU M166N MLS*	This paper
KH402	*ΔrsbPQ ΔrsbTU rsbV-FLAG amyE::ctc-lacZ, pHB201-P(spank)-rsbU M134L MLS*	This paper
KH403	*ΔrsbPQ ΔrsbTU rsbV-FLAG amyE::ctc-lacZ, pHB201-P(spank)- rsbU S167P MLS*	This paper

KH404	*ΔrsbPQ ΔrsbTU rsbV-FLAG amyE::ctc-lacZ, pHB201-P(spank)- rsbU S201G MLS*	This paper
KH405	*ΔrsbPQ ΔrsbTU rsbV-FLAG amyE::ctc-lacZ, pHB201-P(spank)-rsbU S251L MLS*	This paper
KH07	*ΔrsbPQ ΔrsbTU rsbV-FLAG amyE::ctc-lacZ, pHB201-P(spank)-rsbU MLS*	Ho, & Bradshaw 2021
KH406	*ΔrsbPQ ΔrsbTU rsbV-FLAG amyE::ctc-lacZ, pHB201-P(spank)-rsbU M134L MLS*	This paper
KH389	*ΔrsbPQ ΔrsbTU rsbV-FLAG amyE::ctc-lacZ, pHB201-P(spank)-rsbU M166T MLS*	This paper
KH407	*ΔrsbPQ ΔrsbTU rsbV-FLAG amyE::ctc-lacZ, pHB201-P(spank)-rsbU S167P MLS*	This paper
KH408	*ΔrsbPQ ΔrsbTU rsbV-FLAG amyE::ctc-lacZ, pHB201-P(spank)-rsbU S201G MLS*	This paper
KH409	*ΔrsbPQ ΔrsbTU rsbV-FLAG amyE::ctc-lacZ, pHB201-P(spank)-rsbU S251L MLS*	This paper
KH109	*ΔrsbPQ ΔrsbTU rsbV-FLAG amyE::ctc-lacZ, pHB201-P(spank)-rsbU M166V MLS*	Ho, & Bradshaw 2021
KH110	*ΔrsbPQ ΔrsbTU rsbV-FLAG amyE::ctc-lacZ, pHB201-P(spank)-rsbU M166L MLS*	Ho, & Bradshaw 2021
KH330	*ΔrsbPQ ΔrsbTU rsbV-FLAG amyE::ctc-lacZ, pHB201-P(spank)-rsbU M166I MLS*	This paper
KH331	*ΔrsbPQ ΔrsbTU rsbV-FLAG amyE::ctc-lacZ, pHB201-P(spank)-rsbU M166F MLS*	This paper
KH390	*ΔrsbPQ ΔrsbTU rsbV-FLAG amyE::ctc-lacZ, pHB201-P(spank)-rsbU M166N MLS*	This paper

Supplementary Table 2*:*

E. coli strains (BL21 background)

Strain number	Genotype	Reference
SP85	*BL21 (DE3) pET47b-6H-3C-spoIIE-590-827 P752R Kan*	This paper
SP86	*BL21 (DE3) pET47b -6H-3C-spoIIE-590-827 E675S Kan*	This paper
SP87	*BL21 (DE3) pET47b -6H-3C-spoIIE-590-827 I723S Kan*	This paper
SP90	*BL21 (DE3) pET47b 6H-3C-spoIIE-590-827 P752R-E675S-I723S Kan*	This paper
NB1732	*BL21 (DE3) pET47b 6H-3C-spoIIE 590-827 Kan*	Ho, & Bradshaw 2021
NB1959	*BL21 (DE3) pET47b 6H-3C-spoIIAA Kan*	Ho, & Bradshaw 2021
SP12	*BL21 (DE3) pET47b 6H-3C-spoIIAA R67T Kan*	This paper
NB781	*BL21 (DE3) pET47b 6H-3C-spoIIAA spoIIAB Kan*	
NB182	*BL21 (DE3) pET23a 6H-Sumo-spoIIAB amp*	Ho, & Bradshaw 2021
NB1835	*BL21 (DE3) pET47b 6H-3C-rsbV Kan*	Ho, & Bradshaw 2021
